# IL20RB signaling enhances stemness and chemotherapy resistance in pancreatic cancer

**DOI:** 10.1186/s12967-023-04800-5

**Published:** 2023-12-14

**Authors:** Xiao-hui Li, Gui-zhong Huang, Zi-lan Xu, Chong-yu Zhao, Xiao-yuan Dong, Bo-kang Cui, Xiao-jun Lin

**Affiliations:** 1 Department of Pancreatobiliary Surgery, State Key Laboratory of Oncology in South China, Collaborative Innovation Center for Cancer Medicine, Sun Yat-sen University Cancer Center, 651 Dongfengdong Road, Guangzhou, 510060 China; 2 Department of Hepatobiliary Surgery, The Second Affiliated Hospital of Army Medical University, Chongqing, 400037 China; 3 Department of Gynecology, Guangdong Hydropower Hospital, Guangzhou, 510060 China

**Keywords:** IL20RB, Pancreatic cancer, Stemness, Chemotherapy resistance

## Abstract

**Objective:**

Pancreatic cancer is an aggressive malignancy with high mortality, and cancer cell stemness and related drug resistance are considered important contributors to its poor prognosis. The objective of this study was to identify regulatory targets associated with the maintenance of pancreatic cancer stemness.

**Materials and Methods:**

Pancreatic tumor samples were collected from patients at Sun Yat-sen University Cancer Center, followed by immunofluorescence analysis. Pancreatic cancer cell lines with Interleukin-20 receptor subunit beta (IL20RB) overexpression and knockdown were established, and clonal formation, spheroid formation and side population cell analysis were conducted. The effects of IL20RB knockdown on the tumor-forming ability of pancreatic cancer cells and chemotherapy resistance in vivo were explored.

**Results:**

IL20RB expression was significantly upregulated in pancreatic cancer tissues, and was correlated with unfavorable prognosis. The IL20RB receptor promotes stemness and chemoresistance in both in vitro and in vivo models of pancreatic cancer. Mechanistically, IL20RB enhances the stemness and chemoresistance of pancreatic cancer by promoting STAT3 phosphorylation, an effect that can be counteracted by a STAT3 phosphorylation inhibitors. Additionally, Interleukin-19 derived from the microenvironment is identified as the primary ligand for IL20RB in mediating these effects.

**Conclusion:**

Our findings demonstrate that IL20RB plays a crucial role in promoting stemness in pancreatic cancer. This discovery provides a potential therapeutic target for this lethal disease.

**Graphical abstract:**

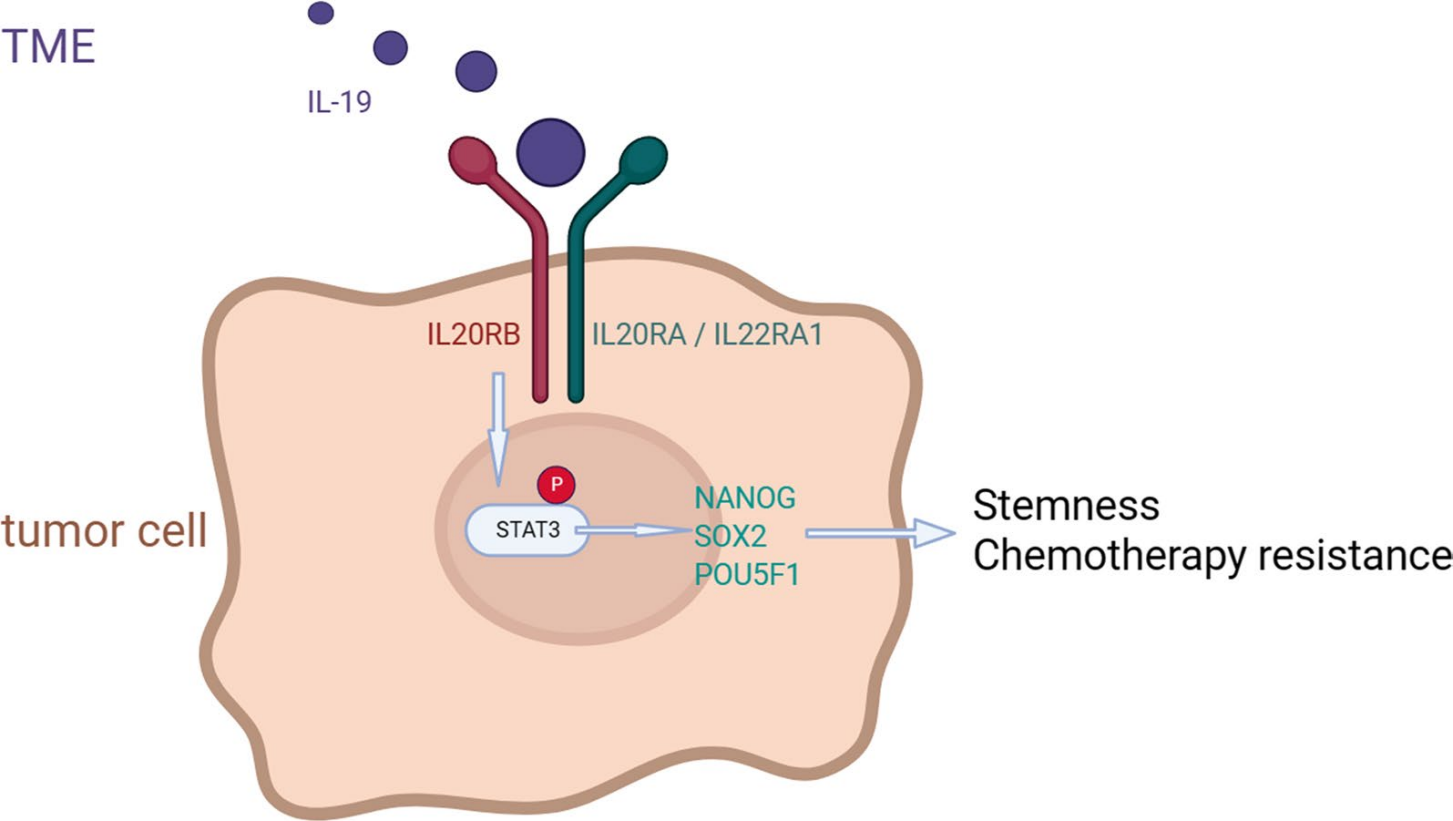

**Supplementary Information:**

The online version contains supplementary material available at 10.1186/s12967-023-04800-5.

## Introduction

Cancer stem cells (CSCs) are a small population of cells in tumors that possess characteristics associated with normal stem cells, such as self-renewal and differentiation. Therefore, CSCs drive tumor recurrence, metastasis, and drug resistance by generating new tumor cells [1, 2]. In addition, CSC also possess a high degree of metabolic plasticity, which allows them to respond to metabolic changes and maintain proliferation, self-renewal and viability [3]. Pancreatic cancer is one of the most aggressive solid malignancies, which has become the fourth leading cause of cancer related deaths in the world [4]. The typical malignant characteristics of pancreatic cancer, such as rapid progression, easy recurrence, and poor drug response, are thought to be related to the strong stemness of pancreatic cancer cells [5, 6]. Given the critical role of stemness in the progression of pancreatic cancer, identification of new targets that enhance the stemness of pancreatic cancer and development of specific therapies targeting stemness may significantly improve the prognosis of pancreatic cancer patients.

The Interleukin (IL)-20 subfamily plays a crucial role among the diverse mechanisms that facilitate stemness. For example, the expression of IL20RA and IL22RA1 promotes stemness in breast cancer [7] and pancreatic cancer [8], respectively. IL-22 has been reported to promote stemness in colorectal cancer [9] and Kras-mutant lung cancer [10]. Interleukin-20 receptor subunit beta (IL20RB) is a subunit of the IL-20 subfamily receptor. It forms a complete heterodimeric receptor with IL20RA or IL22RA1, respectively, and the ligands mainly include IL-19, IL-20 and IL-24 [11]. We found that IL20RB is highly expressed in pancreatic cancer through a previous study by our group, not published online, but the role of IL20RB in pancreatic cancer and whether it promotes pancreatic cancer stemness are unknown.

Here, we examined the correlation between IL20RB expression and clinical characteristics of pancreatic cancer, and explored the underlying mechanisms of how IL20RB modulates pancreatic cancer stemness and chemotherapy resistance. We also evaluated the effect of IL20RB knockdown in mouse models, and the results suggested that IL20RB can be a promising therapeutic target for pancreatic cancer.

## Materials and methods

### Cell culture

The pancreatic cancer cell lines in this study (PANC-1 and MIA PaCa-2) were purchased from the American Type Culture Collection (ATCC). PANC-1 and MIA PaCa-2 cells were cultured using Gibco^™^ Dulbecco's modified eagle medium (DMEM) (Thermo Fisher Scientific, Waltham, MA, USA). The complete culture medium contained 10% fetal bovine serum (FBS, NEWZERUM, New Zealand).

### Western blot test

In short, the proteins in the cells were extracted, quantified, and denatured. Total protein was subjected to SDS-PAGE and transferred to PVDF membranes (Millipore). PVDF membrane was sealed with 5% milk for 1 h at room temperature. In a cool store at 4 ℃, the PVDF membrane was incubated with the first antibody for a whole night. On the second day, the PVDF membrane was incubated with the second antibody at room temperature for 1 h and then exposed to a chemiluminescence instrument to obtain protein bands. Antibodies: rabbit anti-IL20RB antibody (WB: dil. 1:1000, Proteintech, 20,521-1-AP); rabbit anti-NANOG antibody (WB: dil. 1:1000, Proteintech, 14,295–1-AP); rabbit anti-SOX2 antibody (WB: dil. 1:1000, Proteintech, 11,064-1-AP); mouse anti-STAT3 antibody (WB: dil. 1:1000, Cell Signaling Technology, 9139); rabbit phospho-Stat3 (Tyr705) antibody (WB: dil. 1:2000, Cell Signaling Technology, 9145); mouse anti-β-ACTIN antibody (WB: dil. 1:20,000, Proteintech, 66,009-1-Ig) (dil., dilution).

### Real-time quantitative PCR test

Total RNA was extracted from cell lines using Invitrogen^™^ TRIzol reagent (Thermo Fisher Scientific, Waltham, MA, USA). The first strand cDNA was synthesized with random primers by the first strand cDNA synthesis kit (Thermo Fisher Scientific, Waltham, MA, USA). Relative RNA level was determined by real-time quantitative PCR on a Light Cycler 480 II (Roche Diagnostics, Mannheim, Germany) using the SYBR Green method. β-ACTIN was used as an internal control for mRNA levels of IL20RB and associated genes. There were three biological replicates per experiment. The relative expression level of RNA was calculated by the comparative CT method. The sequences of the genespecific primers are listed in Additional file 1: Table S1.

### Plasmids and lentivirus production and transduction

IL20RB overexpression and ShIL20RB knockdown plasmids were successfully constructed in Guangzhou Ruibo Biotechnology Co., Ltd. as target plasmids, and commercially available psPAX2 and pMD2.G as packaging plasmids. The control vector and recombinant plasmids (psPAX2: PMD2.G: IL20RB ratio is 3:1:4) were transfected into 293 T cells to produce lentivirus and infect PANC-1 and MIA PaCa-2 cells, respectively. After 6 h, the supernatant was replaced with a complete medium, and the cells were screened with purinomycin.

### Plate clone formation test

The well-grown log-stage PANC-1 and MIA PaCa-2 pancreatic cancer cells were digested, centrifuged, and re-suspended. They were uniformly distributed in the six-well plate with a density of 2000–4000 cells per well. The cells were incubated at 37 ℃ for 7 to 10 days until the cell colonies were visible to the naked eye. They were stained with crystal violet solution and then photographed. Subsequently, the number of clones formed was calculated using the ImageJ software.

### Sphere-forming experiment

The sphere-forming medium was composed of DMEM/F12 + 1*B27 + 20 ng/ml bFGF + 20 ng/ml EGF. Ultra-low attachment six-well plates (3471, Corning) were selected, and cells were distributed at a density of 2000–5000 cells per well (depending on pelleting efficiency). Each well was supplemented with 4 ml medium. The culture lasted 7–10 days, during which the images were captured by an inverted microscope. In each well, three fields of view were photographed, and the number of cell spheres with a diameter greater than 75 µm was counted.

### Flow cytometry analysis of side population (SP) cells

PANC-1 and MIA PaCa-2 pancreatic cancer cells were inoculated in six-well plates, centrifuged after digestion, suspended in 1 mL PBS, then added with 5 μg/mL Hoechst 33,342, and incubated in a warm box for 60 min. Flow cytometry analysis was conducted for these cells using a Beckman flow cytometer. The ultraviolet laser was used to excite Hoechst 33,342, and the emitted fluorescence signals were detected by the Hoechst Blue and Red fluorescence channels at 450/65 nm and 670/30 nm, respectively. The data were analyzed using FlowJo software.

### Immunofluorescence staining

Pancreatic cancer tissues from patients were embedded into paraffin wax and sliced using a microtome. The slices were roasted, dewaxed with xylene and rehydrated with graded ethanol series. They were immersed in the citric acid solution for antigen retrieval and then treated with 3% hydrogen peroxide to deactivate endogenous peroxidases. The tissues were covered with serum and kept in a wet box at 37 ℃ for 1 h; after that, they were incubated at 4 ℃ overnight in a wet box with a suitable concentration of primary antibodies. Following the addition of second antibodies, the tissues were incubated at room temperature for 1 h away from light. Immediately after the treatment with a DAPI fluorescence-preserving mounting medium, the tissues were observed under a fluorescence microscope. Antibodies: rabbit anti-IL20RB antibody (dil. 1:50, Proteintech, 20,521-1-AP); rabbit anti-NANOG antibody (dil. 1:500, Proteintech, 14,295-1-AP); rabbit anti-SOX2 antibody (dil. 1:150, Proteintech, 11,064-1-AP); rabbit phospho-Stat3 (Tyr705) antibody (dil. 1:300, Cell Signaling Technology, 9145); mouse anti-pan-CK antibody (dil. 1:500, Abcam, ab215838); rabbit anti-IL-19 antibody (dil. 1:500, Bioss, bs-10087R) (dil., dilution).

### Immunohistochemistry (IHC) analysis

Paraffin-embedded tissues of mouse tumors were sliced for this examination. The tissue culture plates were stained with NANOG, SOX2 and pan-CK antibodies. The expression level was evaluated according to staining intensity and percentage of cells stained positive. The staining intensity was negative, weak, medium and strong, respectively, corresponding to scores of 1, 2, 3 and 4. The percentage of cells stained positive was 0–25%, 26–50%, 51–75% and 76–100%, respectively, with scores of 1, 2, 3 and 4. The IHC score was obtained by multiplying the cell percentage score and the staining intensity score. Antibodies: rabbit anti-NANOG antibody (dil. 1:500, Proteintech, 14,295-1-AP); rabbit anti-SOX2 antibody (dil. 1:50, Proteintech, 11,064-1-AP); mouse anti-pan-CK antibody (dil. 1:200, Abcam, ab215838) (dil., dilution).

### Animal experiments

Female BALB/c nude mice aged 4–6 weeks were used for this experiment. They were raised in the Huangpu Experimental Animal Center of Sun Yat-sen University Cancer Center with free access to food and water. The mice (five in each group) were injected subcutaneously with 0.1 ml of cell suspension containing 1 × 10^5^, 1 × 10^6^ or 1 × 10^7^ cells. When the tumor was palpable, its length and width were measured periodically, and the volume was calculated according to the formula: volume = length × width^2^ × 0.5. The animal test of this study was approved by the Ethics Committee of Sun Yat-sen University Cancer Center (Approval Number: L025504202301010).

### Statistical analysis

Results are expressed as mean ± SD for experiments performed in triplicate or more. Student *t*-test was used for statistical analysis of the mean comparison between the two groups. Nonparametric test was used for statistical analysis of abnormal distribution data. Kaplan–Meier method was used for survival analysis. Spearman’s correlations was used to analyze the correlation between IL20RB levels and clinicopathological characteristics. All statistical analyses were performed using SPSS software package (version 25.0; IBM SPSS). *P* < 0.05 was considered significant for all statistical analyses.

## Results

### IL20RB is highly expressed in tumor tissues of pancreatic cancer patients and is associated with poor prognosis

**Fig. 1:**
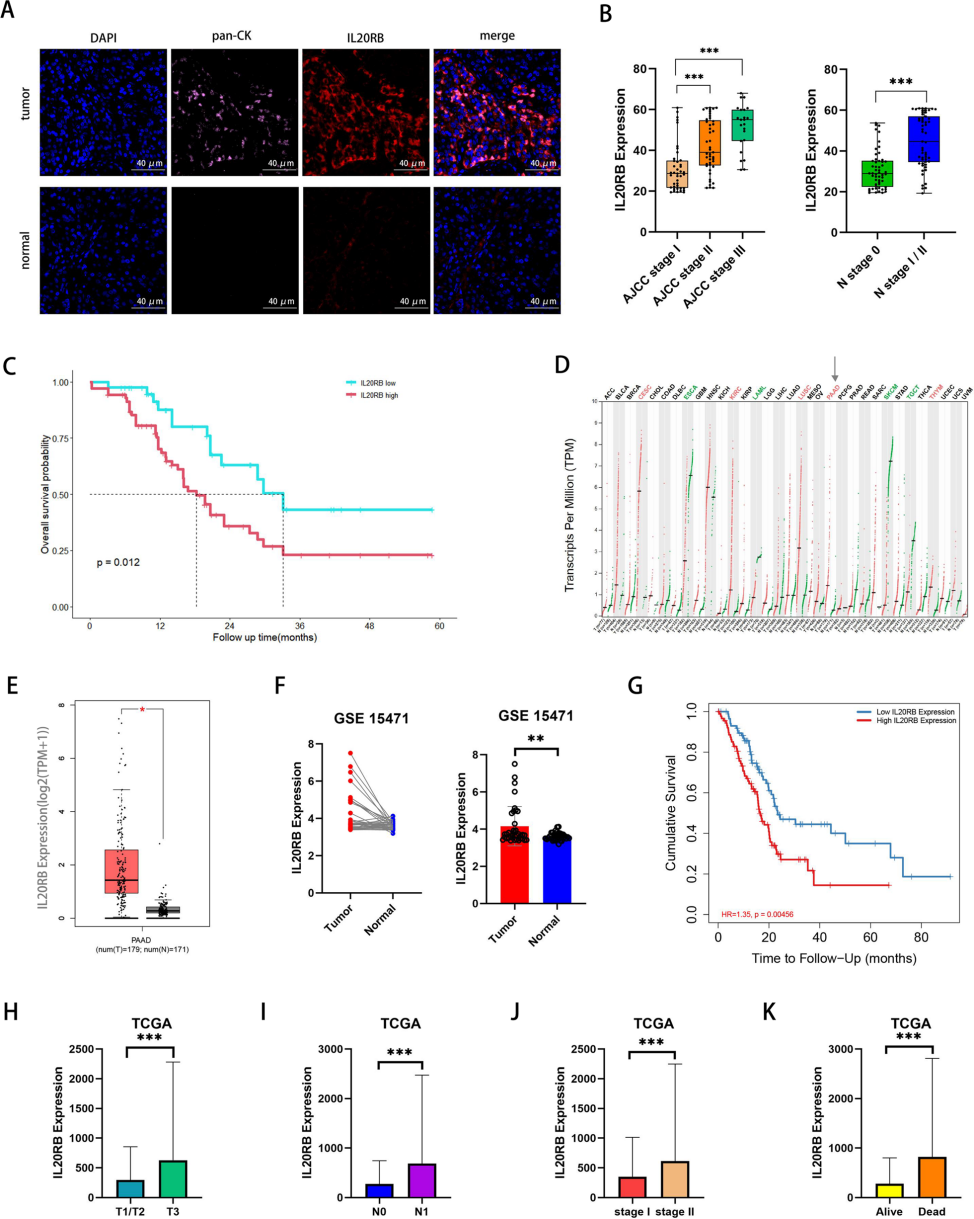
IL20RB is highly expressed in tumor tissues of pancreatic cancer patients and is associated with poor prognosis. **A** Representative immunofluorescence images of IL20RB and pan-CK in human pancreatic cancer sections and surrounding tissues, scale bar = 40 µm. **B** Statistical analysis of IL20RB staining intensity in pancreatic cancer tissue sections from patients at different stages. **C** Correlation analysis between IL20RB staining intensity and overall survival of patients with pancreatic cancer, and the cut-off value was the median of IL20RB staining intensity. **D** IL20RB mRNA expression in pan-cancer and normal tissues, and the red arrow indicates pancreatic cancer, data from TCGA. **E** IL20RB mRNA expression in normal pancreatic tissues and pancreatic cancer tissues, data from TCGA. **F** mRNA expression of IL20RB in pancreatic cancer and paired adjacent tissues, data from Gene Expression Omnibus (GSE15471). **G** Correlation analysis of IL20RB mRNA expression level and overall survival of patients with pancreatic cancer, data from TIMER2.0 database. (**H–K**) Correlation analysis of IL20RB mRNA expression at T stage, N stage and AJCC stage and survival status of patients with pancreatic cancer, data from TCGA

Tumor tissues were collected from 109 patients with pancreatic cancer who had underwent surgical resection at Sun Yat-sen University Cancer Center. Immunofluorescence analysis of tissue sections showed that IL20RB expression was significantly elevated in tumor tissues compared with adjacent normal tissues (Fig. 1A), as well as in advanced stages versus early stages (Fig. 1B). Survival analysis demonstrated that patients with high expression of IL20RB had worse overall survival (Fig. 1C). The Cancer Genome Atlas (TCGA) database showed that IL20RB was highly expressed in multiple cancer types, including pancreatic cancer (Fig. 1D–E). Another publicly available transcriptome dataset GSE15471 also confirmed higher expression of IL20RB in pancreatic cancer than in paired normal tissues (Fig. 1F). In the TIMER2.0 database, shorter overall survival time was observed in IL20RB-high expressing patients compared with IL20RB-low expressing patients (Fig. 1G). In addition, high expression of IL20RB in pancreatic cancer tissues was correlated with late tumor stage (Fig. 1H), late lymph nodes stage (Fig. 1I), late American Joint Committee on Cancer (AJCC) stage (Fig. 1J), as well as patient death (Fig. 1K).

### IL20RB promotes stemness and chemotherapy resistance in pancreatic cancer in vitro

**Fig. 2:**
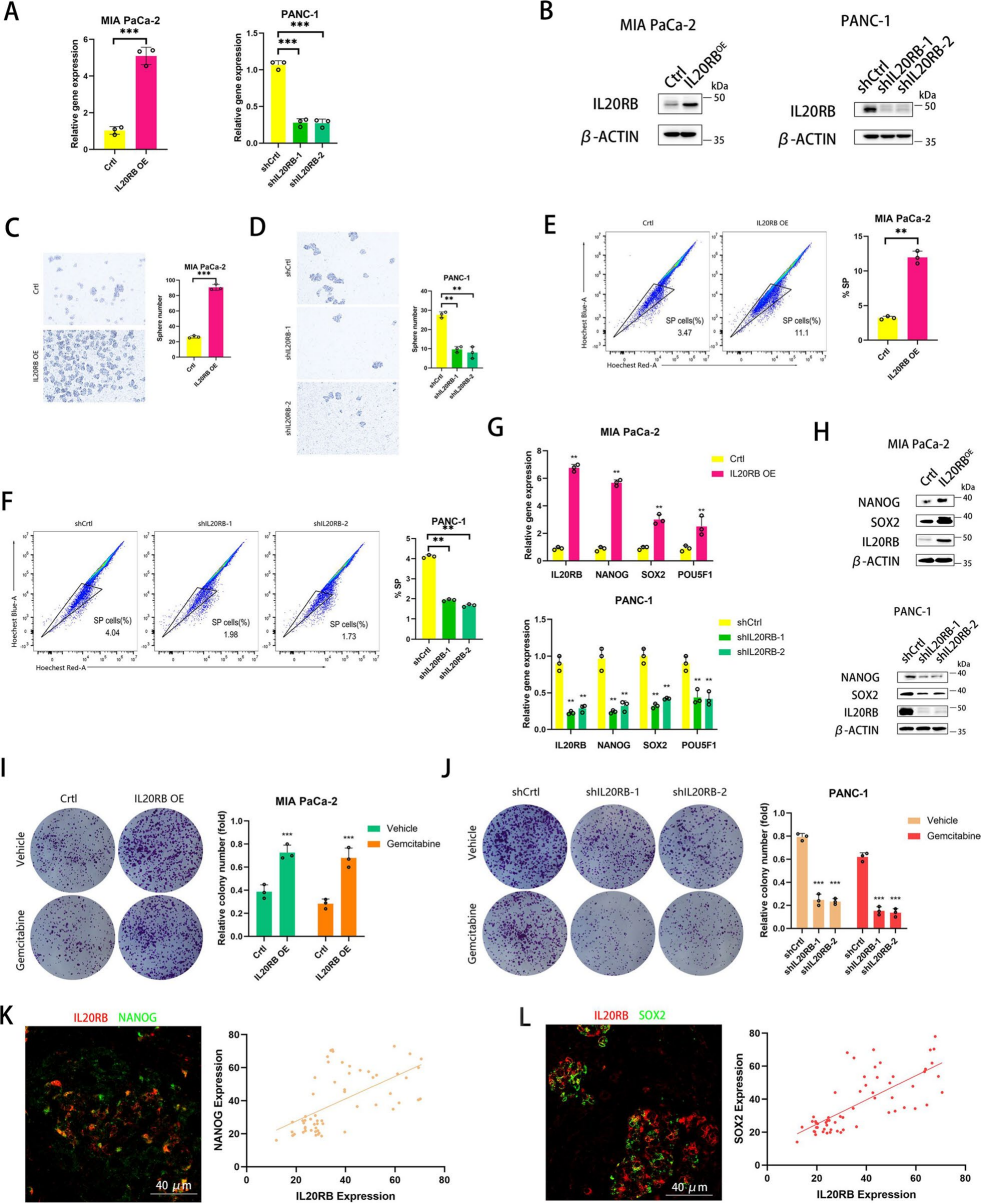
IL20RB promotes stemness and chemoresistance in pancreatic cancer in vitro. **A** Real-time quantitative PCR analysis of IL20RB mRNA expression in pancreatic cancer cell lines PANC-1 and MIA PaCa-2 (n = 3 in each group). **B** Western blot analysis of IL20RB protein expression in PANC-1 and MIA PaCa-2 (n = 3 in each group). **C**–**D** Sphere-forming assay and statistical analysis of PANC-1 and MIA PaCa-2 (n = 3 per group). **E**–**F** Flow cytometry and statistical analyses of side population cells in PANC-1 and MIA PaCa-2 (n = 3 in each group). **G** Real-time quantitative PCR analysis of IL20RB, NANOG, SOX2 and POU5F1 mRNA expression in PANC-1 and MIA PaCa-2 (n = 3 in each group). **H** Western blot analysis of IL20RB, NANOG and SOX2 protein expression in PANC-1 and MIA PaCa-2 (n = 3 in each group). **I**–**J** Results of colony formation assay and statistical analysis of pancreatic cancer cell lines treated with 10 nM gemcitabine for 24 h. **K**–**L** Representative immunofluorescence images and statistical analysis of IL20RB, NANOG and SOX2 in human pancreatic cancer tissue sections, scale bar = 40 µm

Previous studies have reported that some members of the IL20 subfamily promote tumor stemness [7, 8]. In order to explore the potential of IL20RB in enhancing the stemness of pancreatic cancer cells, we constructed stable cell lines, including MIA PaCa-2 with IL20RB overexpression and PANC-1 with IL20RB knockdown (Fig. 2A–B). The spheroid formation experiment showed that pancreatic cancer cells with IL20RB overexpression had significantly more spheroids than the control group, while cells with IL20RB knockdown had significantly fewer spheroids (Fig. 2C–D). SP cell analysis [12, 13] proved that IL20RB overexpression increased the proportion of SP cells, while IL20RB knockdown exerted opposite effects (Fig. 2E–F). Notably, IL20RB overexpression upregulated the mRNA levels of several tumor stemness markers, including NANOG, SOX2 and POU5F1, while IL20RB knockdown downregulated their mRNA levels (Fig. 2G). In agreement with the change at transcriptional level, the protein expression of NANOG and SOX2 were also regulated by IL20RB (Fig. 2H). Collectively, these results suggested that IL20RB promotes the stemness of pancreatic cancer cells.

It has been reported that tumor stemness is closely related to chemotherapy resistance [14, 15]. Here, we found that IL20RB overexpression enhanced the resistance of pancreatic cancer cells to gemcitabine, whereas IL20RB knockdown had the opposite effect (Fig. 2I–J). These results indicated that IL20RB could also promote chemoresistance of pancreatic cancer cells. Subsequently, we performed immunofluorescence analysis for the human pancreatic cancer tissues and found that the expression of IL20RB was positively correlated with the expression of NANOG and SOX2 (Fig. 2K–L). Interestingly, overexpression of IL20RB also promoted the invasiveness of pancreatic cancer compared with control cells in vitro, whereas knockdown of IL20RB had the opposite effect (Additional file 2: Figure S1).

### IL20RB promotes stemness and chemotherapy resistance in pancreatic cancer in vivo

**Fig. 3:**
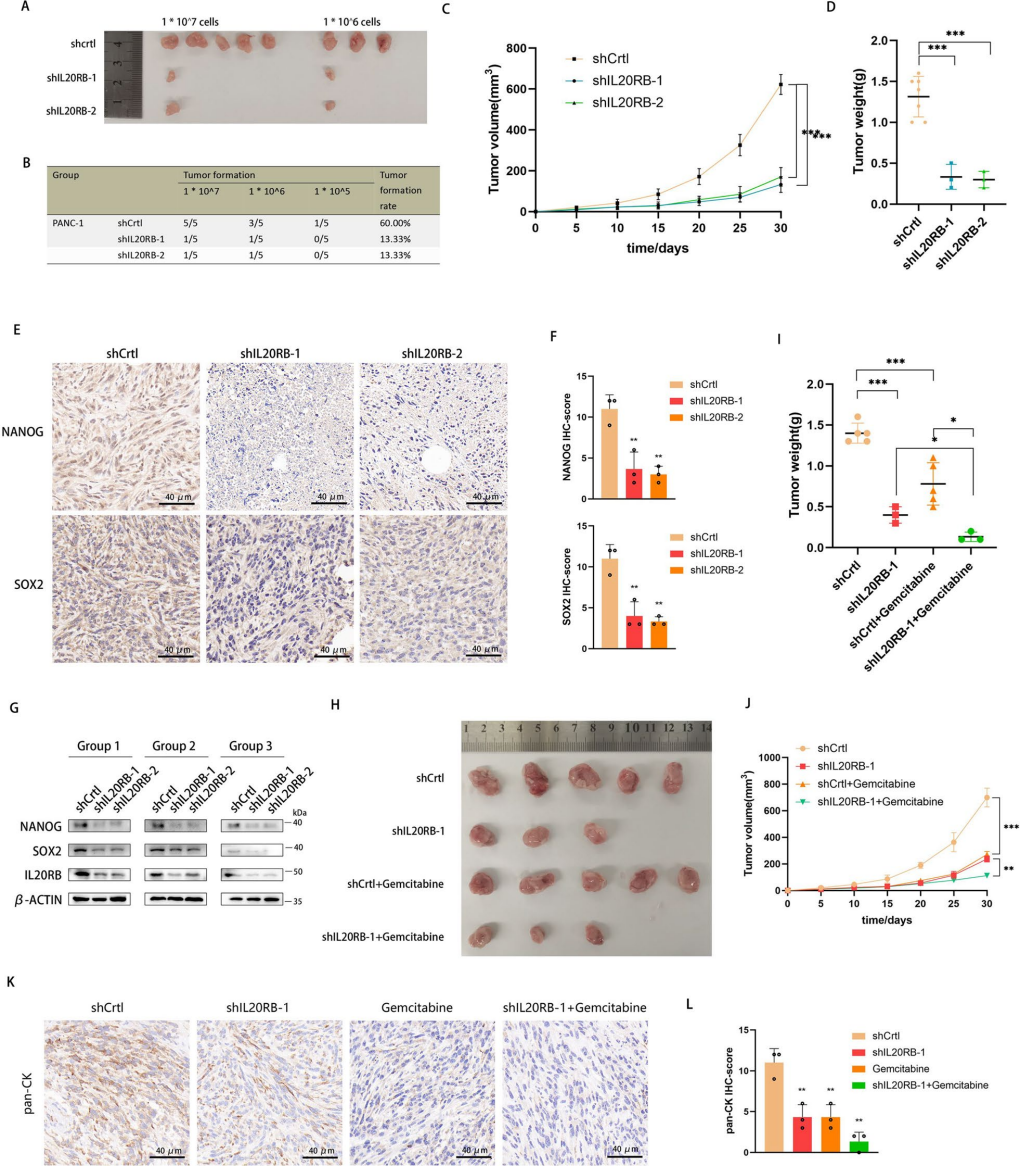
IL20RB promotes stemness and chemoresistance in pancreatic cancer in vivo. **A**–**B** Tumor formation results after subcutaneous injection of 10^5^, 10^6^ and 10^7^ PANC-1, respectively, into nude mice. **C** Tumor growth curve (n = 5 per group). **D** Statistical analysis of tumor weight at the end of the trial (n = 5 per group). **E**–**F** Representative immunohistochemical (IHC) images of NANOG and SOX2 in tumor tissue sections and statistical analysis of IHC scores, scale bar = 40 µm. **G** Western blot analysis of NANOG, SOX2 and IL20RB in tumor tissues. (**H**) 10^7 PANC-1 was injected subcutaneously into nude mice, and then gemcitabine 100 mg/kg was intraperitoneally injected twice a week for 4 weeks. **I** Statistical analysis of tumor weight at the endpoint. **J** Tumor growth curve. **K**–**L** Representative IHC images of pan-CK in tumor tissue sections and statistical analysis of IHC scores, scale bar = 40 µm

PANC-1 cells from the control group and the IL20RB knockdown group were injected into the skin of mice. The tumor formation rate was significantly lower in the IL20RB knockdown group than in the control group (Fig. 3A–B). Moreover, the IL20RB knockdown group displayed significantly smaller tumor volumes and weights than the control group (Fig. 3C–D). IHC of tumor tissue sections showed that the protein levels of NANOG and SOX2 in the IL20RB knockdown group was significantly lower than those in the control group (Fig. 3E–F). Western blot analysis of tumor tissues validated that the protein expression of NANOG and SOX2 was significantly lower in the IL20RB knockdown group than in the control group (Fig. 3G). Notably, IL20RB knockdown combined with gemcitabine treatment further reduced tumor volume and weight compared with IL20RB knockdown alone or gemcitabine treatment alone (Fig. 3H–J). IHC experiments verified that the combination of IL20RB knockdown and gemcitabine treatment further reduced the expression of pan-CK (an epithelial cell marker), compared with IL20RB knockdown alone or gemcitabine treatment alone (Fig. 3K–L). Together, these results suggested that IL20RB promotes the stemness and chemoresistance of pancreatic cancer cells in vivo.

### IL20RB activates the STAT3 pathway to promote stemness and chemotherapy resistance of pancreatic cancer cells

**Fig. 4:**
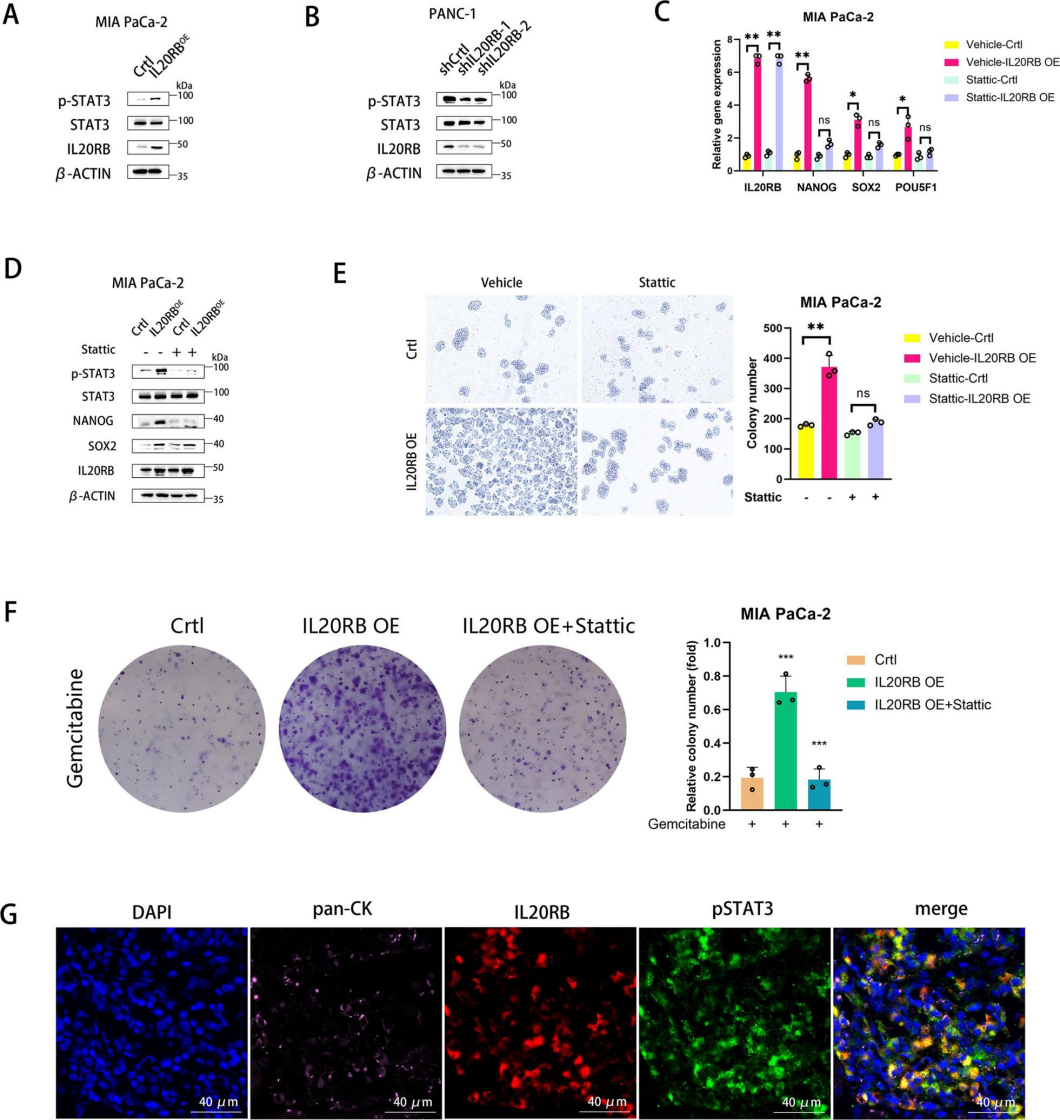
IL20RB activates STAT3 phosphorylation to promote pancreatic cancer stemness and chemotherapy resistance. **A** Western blot analysis of IL20RB, STAT3 and p-STAT3 (Tyr705) proteins in pancreatic cancer cell line MIA PaCa-2. **B** Western blot analysis of IL20RB, STAT3 and p-STAT3 (Tyr705) in pancreatic cancer cell line PANC-1. **C** Real-time quantitative PCR analysis of the mRNA expression of IL20RB, NANOG, SOX2 and POU5F1 in MIA PaCa-2 treated with the vehicle or STAT3 inhibitor (Stattic), respectively (n = 3 in each group). (**D**) Western blot analysis of IL20RB, STAT3, p-STAT3 (Tyr705), NANOG and SOX2 proteins in MIA PaCa-2 after the vehicle or Stattic treatment. **E** Sphere-forming assay and statistical analysis of MIA PaCa-2 after treatment with the vehicle or Stattic (n = 3 per group). **F** Colony formation assay and statistical analysis of MIA PaCa-2 treated with 10 nM gemcitabine for 24 h with or without Stattic (n = 3 per group). **G** Representative immunofluorescence images of IL20RB, pan-CK and p-STAT3 in human pancreatic cancer tissue sections, scale bar = 40 µm

Previous studies have shown that IL-20 subfamily members exert an effect by activating STAT3 (Tyr705) signaling pathway, which plays a crucial role in tumor progression [7, 8, 16]. Western blot experiments verified that the phosphorylation level of STAT3 was upregulated in pancreatic cancer cells overexpressing IL20RB (Fig. 4A) and was downregulated in those with IL20RB knockdown (Fig. 4B). Notably, treating pancreatic cancer cells with Stattic, a STAT3 inhibitor, substantially reversed the upregulation of mRNA expression of tumor stemness markers (NANOG, SOX2 and POU5F1) induced by IL20RB overexpression (Fig. 4C). The effects of IL20RB overexpression on protein levels of NANOG and SOX2 were also restored by Stattic (Fig. 4D). Consistently, the spheroid formation assay showed that the increase in the number of spheroids in cells overexpressing IL20RB was significantly reduced upon Stattic treatment (Fig. 4E). Moreover, STAT3 inhibition reversed the enhanced gemcitabine resistance in IL20RB- overexpressing cells (Fig. 4F). Immunofluorescence staining of the human pancreatic cancer tissues revealed that IL20RB and p-STAT3 were co-expressed in pancreatic cancer cells (Fig. 4G). These results indicated that STAT3 mediates the effects of IL20RB on pancreatic cancer stemness and chemoresistance.

### IL-19 derived from the microenvironment activates the IL20RB-STAT3 pathway to promote stemness and chemoresistance in pancreatic cancer

**Fig. 5:**
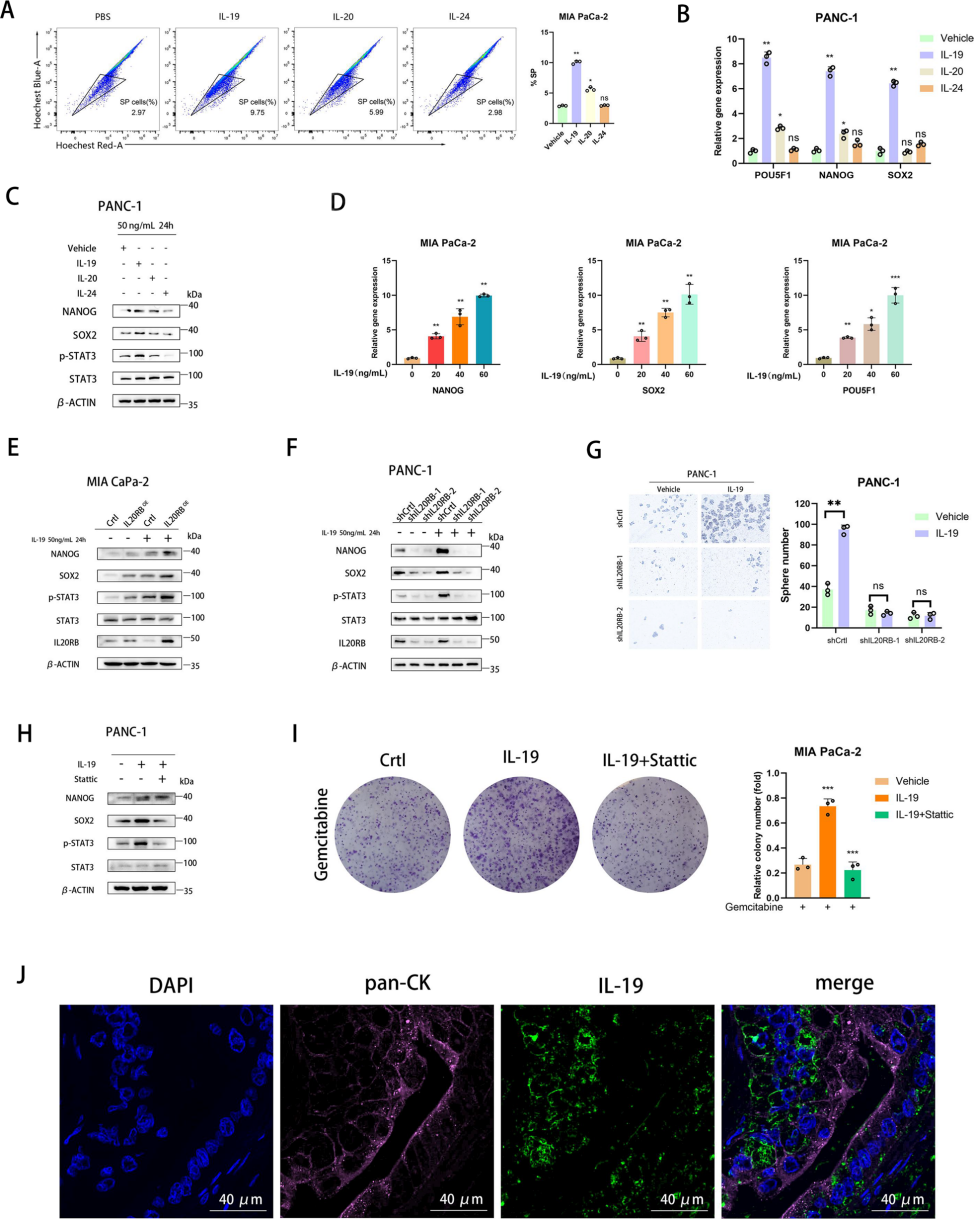
IL-19 derived from the microenvironment activates the IL20RB-STAT3 pathway to promote stemness and chemoresistance in pancreatic cancer. **A** Flow cytometry analysis of side population cells in the pancreatic cancer cell line MIA PaCa-2 after treatment with the ligands of IL20RB (recombinant IL-19, IL-20 and IL-24 proteins) for 24 h and statistical analysis (n = 3 per group). **B** Real-time quantitative PCR analysis of the NANOG, SOX2 and POU5F1 mRNA expression in pancreatic cancer cell line PANC-1 after treatment with recombinant IL-19, IL-20 and IL-24 proteins for 24 h (n = 3 in each group). **C** Western blot analysis of NANOG, SOX2, STAT3, and p-STAT3 (Tyr705) proteins in PANC-1 after treatment with recombinant IL-19, IL-20 and IL-24 proteins for 24 h, respectively. (**D**) Real-time quantitative PCR analysis of the NANOG, SOX2 and POU5F1 mRNA expression in PANC-1 after treatment with 0, 20, 40 and 60 ng/mL recombinant IL-19 protein for 24 h (n = 3 per group). **E**–**F** Results of Western blot analysis of NANOG, SOX2, STAT3, p-STAT3 (Tyr705) and IL20RB proteins in PANC-1 and MIA PaCa-2 after treatment with the vehicle or recombinant IL-19 protein for 24 h. **G** Sphere-forming assay and statistical analysis of PANC-1 treated with the vehicle or recombinant IL-19 protein for 24 h (n = 3 per group). **H** Western blot analysis of NANOG, SOX2, STAT3 and p-STAT3 (Tyr705) proteins in PANC-1 after treatment with the vehicle, recombinant IL-19 protein, or Stattic for 24 h. **I** Results of colony formation assay and statistical analysis of the MIA PaCa-2 after treatment with 10 nM gemcitabine for 24 h, followed by treatment with the vehicle, recombinant IL-19 protein alone or in combination with Stattic (n = 3 per group). **J** Representative immunofluorescence images of IL-19 and pan-CK in human pancreatic cancer tissue sections, scale bar = 40 µm

IL20RB, a cell membrane receptor, whose activation is triggered by corresponding cytokine ligands. Currently known ligands for IL20RB include IL-19, IL-20 and IL-24 [17]. To identify the specific upstream ligand of IL20RB in pancreatic cancer, MIA PaCa-2 and PANC-1 cells were treated with recombinant human IL-19, IL-20 and IL-24 protein. Among them, only IL-19 significantly increased the proportion of SP cells (Fig. 5A), as well as the mRNA and protein levels of stemness markers (Fig. 5B–C). Moreover, IL-19 increased the mRNA expression of stemness markers in a dose-dependent manner (Fig. 5D). IL20RB overexpression significantly increased the protein levels of stemness markers and p-STAT3, and this effect was further enhanced upon IL-19 treatment (Fig. 5E). Consistently, IL20RB knockdown substantially reversed IL-19-induced upregulation of stemness markers and p-STAT3 (Fig. 5F). Spheroid formation assay confirmed the significant increase in the number of spheroids induced by IL-19, which was counteracted by the knockdown of IL20RB (Fig. 5G). In addition, the ability of IL-19 in promoting the expression of stemness markers and p-STAT3 was largely abolished on Stattic treatment (Fig. 5H). Similarly, IL-19 enhanced chemotherapy resistance in pancreatic cancer cells, and such an effect was reversed by Stattic (Fig. 5I). IL-19 has been reported to be secreted by immune cells [18, 19]. Here, we verified the presence of IL-19 in the microenvironment of human pancreatic cancer by immunofluorescence staining (Fig. 5J). Collectively, these findings suggested that microenvironment-derived IL-19 activates IL20RB-STAT3 pathway to promote the stemness and chemoresistance of pancreatic cancer cells.

## Discussion

Cancer stemness refers to the characteristics of CSCs capable of proliferation, self-renewal and multidirectional differentiation [1, 2]. Finding the signaling pathways regulating cancer stemness is crucial for the treatment of cancer. Previous studies have found that the oncogene MYC drives stemness in breast and pancreatic cancer [20, 21]. Cytokines in the tumor microenvironment (TME), such as TGF-β1 and IL-6, have also been reported to promote the stemness of various cancer cells [22, 23].

IL20RB is expressed in a variety of normal cells, including keratinocytes, fibroblasts, monocytes, T cells and endothelial cells [11]. High expression of IL20RB was reported in lung cancer bone metastases [24], clear cell renal cell carcinoma [25] and papillary renal cell carcinoma [26]. In this study, we found that IL20RB was highly expressed in pancreatic cancer samples and correlated with poor prognosis. Increasing studies purported the potential of IL-20 subfamily members in promoting cancer stemness. For example, it has been demonstrated that IL-22 acts on colorectal cancer cells to promote the activation of transcription factor STAT3 and expression of histone H3 lysine 79 methyltransferase, consequently increasing cancer stemness and tumorigenic potential [9]. IL22RA1 has been reported to promote the stemness and tumorigenicity of pancreatic cancer cells by activating STAT3 [8]. In the present paper, we found a positive correlation between the protein expression of stemness markers (NANOG and SOX2) and IL20RB in pancreatic cancer samples, suggesting that IL20RB may promote tumor stemness. We also found that IL20RB overexpression in pancreatic cancer cells increased the number of spheroids, the proportion of SP cells and the expression of stemness markers, indicating that IL20RB resulted in stronger stemness. The in vivo studies further confirmed that IL20RB enhanced the tumorigenic ability of pancreatic cancer cells. Tumor stemness is an important factor leading to chemoresistance in pancreatic cancer [27]. Drug resistance is thought to be an intrinsic property of normal stem cells and CSCs and is acquired through multiple independent mechanisms, such as the upregulation of drug efflux pumps, superior DNA repair capacity, or enhanced protection against Reactive Oxygen Species [28–30]. To further understand whether IL20RB regulates chemotherapy resistance, we performed clone formation assays on gemcitabine-treated pancreatic cancer cells and found that IL20RB overexpression enhanced drug resistance; in contrast, knockdown of IL20RB weakened drug resistance in pancreatic cancer cells. The in vivo study also confirmed that IL20RB knockdown combined with gemcitabine treatment further reduced tumor volume and weight.

IL20RB exerts its effects by binding to IL-19, IL-20 and IL-24 [11, 31]. IL-19 and IL-20 were identified as IL-10 homologs in the expressed sequence tag database [18, 32]. IL-24 was detected in terminally differentiated human melanoma cells induced by interferon β and the protein kinase C activator mezerein [33]. It has been reported [34, 35] that the major source of IL-19, IL-20 and IL-24 is myeloid cells, followed by epithelial cells. We found that IL-19 most significantly increased the proportion of SP cells and the expression of stemness markers in pancreatic cancer cells in vitro, indicating that IL-19 plays a major role in promoting stemness in pancreatic cancer. Immunofluorescence analysis confirmed the presence of IL-19 in the TME of clinical pancreatic cancer samples, which is consistent with findings in previous studies [18, 19], suggesting the mechanism by which TME factors promote the stemness of pancreatic cancer cells. Moreover, IL20RB knockdown reversed the IL-19-mediated spheroid augmentation and upregulation of stemness markers, indicating that IL20RB is responsible for the effect of IL-19.

The STAT protein family include STAT1, STAT2, STAT3, STAT4, STAT5 (STAT5A and STAT5B) and STAT6 [36]. STAT3 is involved in the proliferation of tumor cells, inhibition of apoptosis and promotion of stemness and chemotherapy resistance in cancer cells. STAT3 over-activation induces immunosuppression and tumor invasion [37–39]. In this sense, STAT3 has emerged as a promising target in cancer treatment. In the present study, we found that the STAT3 signaling pathway played an important role in mediating the function of IL20RB in promoting cancer stemness and chemoresistance, and IL20RB-STAT3 signaling promoted the expression of NANOG, SOX2 and POU5F1. In addition, the co-expression of IL20RB and PSTAT3 was observed in clinical pancreatic cancer samples.

However, this work has some limitations. Firstly, the mechanism by which IL20RB exerted its effects was explored only in vitro. Secondly, the specific cells producing IL-19 in the TME need to be further investigated. Thirdly, this study lacks therapeutic experiments targeting IL20RB, and the effectiveness of pancreatic cancer therapies targeting IL20RB requires verification.

In conclusion, the present study is a pioneering work probing into the role of IL20RB in pancreatic cancer. IL20RB was found to be highly expressed in pancreatic cancer and enhanced the stemness properties of pancreatic cancer cells and confer resistance to chemotherapy. Mechanistically, this effect is mediated through the activation of the downstream STAT3 pathway by IL20RB. Notably, microenvironment-derived IL-19 serves as the primary ligand initiating the signaling cascade mediated by IL20RB in pancreatic cancer.

### Supplementary Information

**Additional file 1: Table S1.** List of primer sequences for PCR experiments.

**Additional file 2: Figure S1.** IL20RB promotes pancreatic cancer invasiveness in vitro. Transwell assay and statistical analysis of PANC-1 and MIA PaCa-2 (n=3/group).

## Data Availability

The data used to support the findings of this study are included within the article.

## Abbreviations

IL: Interleukin
CSCs: Cancer stem cells
ATCC: American type culture collection
DMEM: Dulbecco’s modified eagle medium
SP: Side population
IHC: Immunohistochemistry
TCGA: The Cancer Genome Atlas
AJCC: American Joint Committee on Cancer
TME: Tumor microenvironment
