# Supplementary material for: IL20RB signaling enhances stemness and chemotherapy resistance in pancreatic cancer
Source: J Transl Med. 2023 Dec 14;21:911. doi: 10.1186/s12967-023-04800-5 (PMC10722837; doi:10.1186/s12967-023-04800-5)
Supplement: Supplementary file 2 — Additional file 2: Figure S1. IL20RB promotes pancreatic cancer invasiveness in vitro. Transwell assay and statistical analysis of PANC-1 and MIA PaCa-2 (n=3/group). [file 12967_2023_4800_MOESM2_ESM.pdf]

Additional file 1: Figure S1

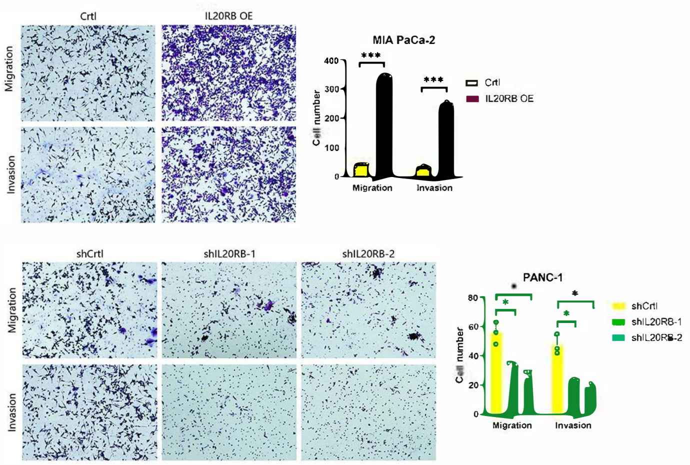

Supplementary Figure 1:IL20RB promotes pancreatic cancer invasiveness in vitro. Transwell assay and statistical analysis of PANC-1 and MIA PaCa-2 (n=3 / group).
